# Patient Satisfaction with Private Recovery Services and Importance of Physician Behavior during COVID Time

**DOI:** 10.3390/healthcare9080928

**Published:** 2021-07-23

**Authors:** Ecaterina Coman, Alexandru Diaconu, Luiza Mesesan Schmitz, Angela Repanovici, Mihaela Baritz, Claudiu Coman, Silvia Fotea

**Affiliations:** 1 Department of Management and Economic Informatics, Faculty of Economic Science and Business Administration, Transilvania University of Brasov, 500036 Brașov, Romania; ecaterina.grajdieru@unitbv.ro; 2 Department of Fundamental and Prophylactic Disciplines, Faculty of Medicine, Transilvania University of Brasov, 500036 Brașov, Romania; diaconualexandru96@yahoo.com; 3 Department of Social and Communication Sciences, Faculty of Sociology and Communication, Transilvania University of Brasov, 500036 Brașov, Romania; luiza.mesesan@unitbv.ro (L.M.S.); claudiu.coman@unitbv.ro (C.C.); 4 Department of Product Design, Mechatronics and Environment, Faculty of Product Design and Environment, Transilvania University of Brasov, 500036 Brașov, Romania; mbaritz@unitbv.ro; 5 Clinical Medical Department, Faculty of Medicine and Pharmacy, “Dunarea de Jos” University, 800008 Galati, Romania; silvia.fotea@ugal.ro

**Keywords:** patient satisfaction, medical recuperation services, physician’s behavior, recuperation clinic

## Abstract

Introduction: Patient satisfaction represents an essential indicator of the quality of care in the medical recuperation sector. This study aimed to identify the degree of satisfaction in patients who benefit from medical recuperation services in one private clinic from Romania and the factors that played a part in this respect. Method: An online questionnaire was completed by 105 patients of a private clinic in the period immediately following the opening of the clinic after the quarantine period due to COVID-19. The following concepts were measured: general satisfaction with clinical recuperation services (SG), physician’s behavior (PB), the impact of interventions on the state of health (IHI), modern equipment (ME), and the intention to return to the clinic (IRC). Based on a linear regression model, the impact of PB, IHI, ME, and IRC variables on general satisfaction (SG) was established. Results: The study results confirm the data from studies carried out in different sociocultural contexts in ordinary time, where physician behavior is the most crucial factor in patients’ satisfaction. Therefore, we can say that the physiotherapist’s behavior has an essential role in determining the patients’ satisfaction both in ordinary time and in COVID-19 time. The data in this study reflect the fact that satisfaction with the services offered by a medical recuperation clinic is a predictor for using the services in the future. Still, our study reflects a moderate relationship in intensity.

## 1. Introduction

Since 1986, the World Health Organization has considered health as a resource of daily life and not an objective of existence. Medical recuperation services, by both their preventive and corrective role, illustrate a premise more and more important in reaching this aim, namely social and economic reintegration of their beneficiaries despite the difficulties resulting from the disease [1]. Within a European context, according to the data reported within the Eurobarometer, especially for physical activity, published in 2018, Romania, with 63%, holds fourth place after Bulgaria, Greece, and Portugal at 68%, being among the 11 countries in the EU in which more than half of the population never takes physical exercise or does sport [2].

These data corroborated with the fact that healthy life expectancy at birth is almost constant in time, and if life expectancy at birth increases, it leads to a more considerable period in which people live with various disorders, including those that pertain to medical recuperation specific especially to people advanced in age.

Within this context, with an increasingly greater need for medical services, the medical system in Romania progressed, especially by developing private medical units. In recent years, the number of private medical institutions has increased, while the number of the public hospitals has fallen [3]. However, the access to rehabilitation services covered by health insurance is inadequate, with long waiting lists, ranging from several months to two years [3]. Thus, people who care about their health often find themselves forced to pay for these services and choose those offered by private medical recovery clinics, especially during the pandemic caused by the SARS-CoV-2 virus.

Patients prefer to visit private hospitals because of better interaction with the staff, latest technology, greater attention that the doctor pays to the patients’ needs, longer duration of consultation, and well-explained procedures [4]. In Romania, the average duration of consultation in the public healthcare sector is 11–20 min, while, in private clinics, it is 21–30 min [5]. Romanian studies on patients’ satisfaction show that the quality of attention and care positively influences the level of satisfaction even when the healthcare resources are limited [6,7]. Therefore, physician behavior can compensate for shortages of healthcare resources and contribute to increasing overall patient satisfaction in both private and public clinics. However, in the public healthcare centers, the efficiency of the therapist–patient communication is unsatisfactory, which determines many of the patients to choose the services of private clinics. The analysis of the satisfaction of patients who use the services of such clinics is more than useful, representing a major indicator of the quality of care in the medical recuperation sector, and it can be the basis for carrying out an appropriate and efficient strategy for the improvement of these services. Satisfaction influences patients’ choice of physician and leads to more excellent patient retention and a greater desire to engage in word-of-mouth [8], thus increasing the attractiveness of a particular healthcare service [9]; thereby, the clinic can obtain a sustainable competitive advantage.

Starting from the abovementioned, in this study, we wished to analyze if, in the Romanian context, the physician’s behavior has an essential role in determining the satisfaction or other variables, as the results of an intervention or modern equipment have a more significant impact on the general satisfaction with the services offered by the clinic. At the same time, it is interesting to compare the results of this study conducted during the pandemic with the results of other similar studies conducted in ordinary time to see if the importance of the physician’s behavior in determining patient satisfaction is influenced by the health crisis or not. COVID-19 time may be relevant given that the COVID-19 safety measures undertaken by the clinic may affect the physician’s behavior and the overall patient satisfaction. The rest of the paper is presented as follows. Section 2 presents a relevant review of the literature that highlights the relationship between patient satisfaction and certain behaviors specific to physicians. Research questions are also offered in this section. Section 3 explains the research methodology: data collection and participants, measurement tools, and data analysis method. Section 4 presents the statistical analysis and results. Section 5 discusses the results. The last section concludes our research, suggests some practical implications for institutions providing such services, and presents the limitations of the study and future research recommendations.

## 2. Background

The polls regarding patient satisfaction, in essence, assess the provision of medical services based on the patients’ point of view. The level of satisfaction is determined by the evaluation of the congruence between the overall healthcare experience and patients’ needs, values, and expectations [10]. Through analysis in the most essential bibliometric databases—The Web of Science, PubMed, Scopus, ProQuest, Emerald, Wiley Online Library, Science Direct, Cochrane Library, and Embase databases—the variables that generate the greatest impact on patient satisfaction were identified.

Research findings on this topic suggest that patient satisfaction is a multidimensional construct influenced by clinical outcomes, physiotherapist features, patient features, physiotherapist–patient relationship, treatment features, and healthcare-setting features [11]. Ng and Luk [12] analyzed the concept of “patient satisfaction” based on a literature review and identified four attributes of patient satisfaction in the healthcare context: provider attitude, technical competence, accessibility, and efficacy. Researchers synthesize the attributes of this concept differently, but the key elements are similar. The studies carried out in other sociocultural contexts indicate that the physician’s behavior has an essential role in establishing the degree of satisfaction with the services provided and, indirectly, with the respective clinic [11]; the interpersonal and technical attributes of the physiotherapist [13,14]; and the process of healthcare being key determinants of patient satisfaction [15]. In most cases, the healing effect of the patient’s reliance on a physiotherapist is more important than the recommended treatment [16].

As evidenced by the literature, all medical staff contribute equally to patient satisfaction, and this satisfaction contributes to the well-being and progress in the recuperation and healing process [17,18]. Physicians, nurses, and auxiliary staff interact with patients, and the impact is significant. Among the medical staff, physiotherapists play a unique role. Regarding the physiotherapist profession, his/her responsibility is based on professional autonomy and clinical practice based on proofs. In order to preserve professional independence, establishing and observing an ethical component is crucial [19]. In this respect, various subjects were tackled and analyzed, such as the optimal way of carrying out the first session, the influence of the clinical environment when starting the first session, and what the beneficial actions for a patient are at the first session. Results prove that, in the case of private practice, the first session has special importance. This moment has a significant role in the physiotherapist–patient relationship. Establishing a social framework and a clinical context with an ethical approach has generated positive results. Within private clinics, the patient’s satisfaction and trust have had high values [4,20]. Patient confidence in the physician, good communication skills, and information provision on the part of the physician predict patient satisfaction [21].

Previous studies noted that the satisfaction of patients correlates with the self-perceived health of patients, with the results obtained following the interventions but also with the probability of returning to the provider for long-term care [22]. Other studies indicate a positive relationship between the degree of satisfaction of patients with the services offered by a clinic and the degree of satisfaction of patients with the physician–patient relationship, and this is high according to the results of the intervention upon the state of health [23,24]. Considering the positive association between the quality ratings of healthcare institutions and physicians [25] and the impact of patient satisfaction on financial performance [26], in some institutions, patient satisfaction is a critical component of physicians’ ranking and reward [27,28]. Therefore, satisfaction with medical services is one of the essential factors that determines the success of an institution providing medical services, and physicians should be directly interested in improving interaction with patients.

Authors such as Daei [29] have sought to obtain a comprehensive understanding of a physician’s behavior, looking for specific elements that can improve patient satisfaction. When we refer to a physician’s behavior, there are certain specific elements that influence patient satisfaction, such as communication and interpersonal skills (encouragement, confidence, empathy, friendly manners, and nonverbal communication); therapeutic skills of physicians (therapist expertise, professional knowledge, and ability to provide good education); individualized patient-centered care (careful examination of patients, individualized treatments) [12,13,16]. The ability to provide information about the problem, the treatment plan, expected treatment effects, what the patient could do to help himself/herself, and decision-making are also very important [30].

Nonverbal behavior was another variable taken into account as a possible predictor of patient satisfaction. This can facilitate a better understanding of interactions between a clinician and patient during the integrative medicine visits, and it can offer information in order to improve clinical interactions in conventional medicine [31]. Another variable with an impact on patient satisfaction was the characteristics of a physician’s voice. A total of 35,597 medical service voices were analyzed, provided by physicians in a mobile health community. Results show that patient satisfaction is positively influenced by direct discussions with the physician but negatively influenced by the case in which the patient receives consultations by means of a voicemail center or a machine with recorded messages. A quick speaker and neuronal emotion utterances are more liable to be associated with higher satisfaction of a patient than a slow speaker and utterances with low and high emotional states. In any case, these effects are weak for the physicians with a high professional capital, which implies that the voice has a substitute role for the quality of services provided [32].

Another characteristic of physicians that was analyzed was their self-esteem. The study has proved that physicians with low self-esteem obtained a lower score regarding trust and anticipated satisfaction than physicians with high self-esteem. The results of this study highlight the way in which a physician’s self-esteem can affect the care process even before its start. High self-esteem generates high patient satisfaction [33].

On the basis of revising the literature in the PsycINFO1 and MEDLINE1 databases, Carrard proposes a behavior adaptability model (PBA) with the purpose of inspiring new research. The PBA model is based on the fact that the physician adapts his/her behavior depending on the different preferences of patients. The PBA model proves how physicians infer the preferences of patients and adapt their interactions from one patient to another. The study proves that the patients will benefit from better results if their physicians present this behavior adaptability rather than an approach “of unique dimensions” [34] because patients appreciate more the care providers who treat them as an individual [30].

The relationship between the patient and the therapist can also be influenced by organizational factors, such as waiting time and time provided for consultation [13,35]. Studies show that actual waiting times have a negative impact on overall patient satisfaction [36]. However, patients’ satisfaction can be increased by turning waiting time into attention received by patients [7]. On the other hand, time spent with physician has a positive effect on overall patient satisfaction. Moreover, the patient who is highly satisfied with the duration of the consultation is 94 percent likely to engage in word of mouth [37].

It is proved that the payment methods preferred by patients, namely the collaboration of the physician with the health insurance fund, and, in this case, some expenses are covered for the insured patients, radically influence the relationship with patients. These models influence the perception upon the quality of medical services and improve the patients’ trust [38]. The relationship of the employer and the private clinic with the medical staff regarding their payment method was also analyzed. Differential pay depended on the specialization of the medical staff; clinical results generate an impact on the perception of the quality of the medical intervention [36,37].

Patients’ satisfaction with the results obtained following an intervention can be subjective or objective. The personal interpretation is based on their experience and perceptions of treatment outcomes, while the objective interpretation is linked to professional evaluation of the outcomes of the intervention [39]. A systematic literature review of 35 studies highlights the positive association of the physician communication behavior with patient satisfaction, as well as with improved health outcomes [17]. Moreover, higher levels of trust in the physician and active patient participation in decision-making were linked to better global health, greater treatment satisfaction [40], and patient adherence [18].

Patient satisfaction can be influenced by physical evidence that the clinic will provide quality services. Thus, tangible elements, such as cleanliness, the availability of modern equipment, and the feeling that the facilities of the clinic are in good condition, can increase overall satisfaction [41]. The availability of newer and safer equipment may positively influence the relationship with physician [42]. The lack of modernity of medical equipment can have a negative impact on overall satisfaction [8,30]. Equipment that is broken or does not work in optimal conditions does not allow participation in treatments or may cause injury [43]. However, a study conducted in Australia and Korea revealed that patient satisfaction with musculoskeletal physiotherapy care is influenced more by “person-focused care” than physical aspects of care, such as up-to-date equipment. For both cultures, overall patient satisfaction was significantly but weakly correlated with the results obtained following an intervention [44].

Few studies were conducted in Romania on patient satisfaction topic and are focused more on assessing patient satisfaction with public health services [3,7,45,46,47,48] and the influence of patient features to satisfaction [5,6] conducted a study to identify the differences between the physician–patient relationship in the private and public healthcare sectors from the point of view of physicians. The answers were similar to the questions related to patient satisfaction, feedback, and communication with patients. There were some differences regarding patient loyalty strategies and attention paid to patients (duration of consultations and benefits offered to patients), which were more critical in the private institutions. Cosma et al. [49] identified that one-third of the patients were unsatisfied with the quality of the Romanian national health system. The facilities of public hospitals were perceived by the respondents as unsatisfactory, except for medical equipment, which was positively appreciated by the majority.

Regarding the rehabilitation services in Romania, the quality of services is higher in the private system due to the availability of the latest treatments, respect for patient privacy, and the initial evaluation of optimal conditions, and a better patient–physiotherapist relationship [50].

As other studies show, the physician’s behavior also has an essential role in establishing the degree of satisfaction with the services provided indirectly by the respective clinic [7,18,39,51]. This research aimed to identify the degree of satisfaction in patients benefitting from medical recuperation services from a private clinic in this field in Romania and the factors that played a part in this respect. We wished to see whether this satisfaction of patients is determined mainly by a physician’s behavior, or rather by results obtained following an intervention or by modern equipment owned. At the same time, we wished to see if the satisfaction with the services offered by the clinic is a predictor of the intention to return to the clinic, as it resulted from other studies.

Therefore, we carried out an inquiry based on a questionnaire to answer the following research questions:

1. What was the impact of the physician’s behavior, the state of health following the intervention, and the modern medical equipment on the general degree of satisfaction with the services offered by the clinic?
2. To what extent does the satisfaction with the services offered by the clinic predict the intention of returning to the recuperation clinic?

## 3. Materials and Methods

We conducted a quantitative study to identify the degree of satisfaction in patients who benefit from private medical recuperation services in Romania and the factors that played a part in this respect in the period immediately following the opening of the clinic after the long quarantine period due to COVID-19, June/July 2020.

### 3.1. Data Collection Method and Participants

Data were collected online by means of the SurveyMonkey platform during June/July 2020. The approval of the institution providing the medical recuperation services and the informed consent signed by the participants were obtained, clinical VITALMED CENTER, Brasov, Romania, approval no. 558/4 April 2020.

The population for the study was selected in a non-probabilistic way. The private clinic was lockdown in the period March–May 2020 due to the pandemic. After the clinic resumed its activity, the number of patients was lower than usual given the protection measures imposed by the pandemic, as well as the fear of the population becoming infected with the new virus. Consequently, the sample size was small, consisting of 105 patients who used the private services of the clinic when it was reopened. Subsequently, the questionnaire was sent as a link to their e-mail addresses. The questionnaire was self-administered, and the average time needed to answer the questionnaire was 15 min.

A majority of the participants were women (58.1%), were up to 60 years old (46.7%), and were married (69.5%). The more significant part of respondents completed high school (47.6%) and had average incomes below the medium average wage (1500–3000 RON) (Table 1).

Regardless of their level of studies or income, the need for medical recuperation is a priority, and these persons were willing to appeal to private medical recuperation services even if the disorders they were suffering from belonged rather to the least severe category (Table 2). Most persons came for lumbar discopathy (25.7%), spondylosis (16.2%), or gonarthrosis (9.5%), these being also very frequent disorders among the population.

### 3.2. The Research Instrument

For data collection, a standardized questionnaire was used. The questionnaire included items corresponding to the two research questions. The following concepts were measured: general satisfaction with clinical recuperation services (SG), physician’s behavior (PB), the impact of interventions on the state of health (IHI), modern equipment (ME), the intention to return to the clinic (IRC). We pre-tested the questionnaire on 24 patients and rephrased some items identified as too challenging to follow.

#### 3.2.1. General Satisfaction with the Clinical Recuperation Services

To measure the general satisfaction with the clinical recuperation services, the following question was asked “On the whole, how satisfied are you with the services received in this clinic? Give a mark from 1 to 10, where 1 not satisfied at all, ten very satisfied”.

#### 3.2.2. Physician’s Behavior

To measure physician’s behavior (PB) an eleven-item scale (Cronbach’s Alpha = 0.86) was used, which reiterates the question, “The therapist did/was…?” for eleven elements related to the activities of the therapists (three categories: 2—He did this as he should, 1—He did this to a certain extent, and 0—He did not do this at all). A summative index was computed, ranging from 0 to 22, where 0 = he did not do any of the 11 actions, and 22 = he performed all 11 activities very well.

#### 3.2.3. Changes in the State of Health after the Intervention

Changes in the state of health after the intervention *(CHI)* were measured by two variables. They assessed one’s own state of health before the intervention (HBI) and assessed one’s own state of health after the intervention (HAI). HBI was measured by using the following question: “How would you evaluate your state of health before coming to the medical recuperation center? Give a mark from 1 to 10, where 1 very poor, 10 very good”. HAI was measured by using the following question: “How would you evaluate your state of health after coming to the medical recuperation center? (HBI) Give a mark from 1 to 10, where 1 very poor, 10 very good”. CHI was measured by the difference between HBI and HAI, ranging from 0 to 6, where 0 is no change and 6 is very great change.

#### 3.2.4. Modern Equipment

To measure modern equipment (ME), the following question was asked: “How satisfied are you with modern equipment?” (5-point Likert scale, where 1 = very dissatisfied, and 5 = very satisfied). Patients evaluate the modern equipment of the recovery clinic based on their perception that the equipment is new, safe, and in good condition.

#### 3.2.5. Intention to Return to the Clinic

To measure the intention to return to the clinic (IRC), the following question was asked: “Would you return to the medical recuperation clinic?” The following response options were offered: “2—certainly yes”, “1—probably”, and “0—certainly no”, out of which a dummy variable was created, the response option “certainly yes”, taking the value 1, and the others the value 0.

The final part of the questionnaire contained a series of sociodemographic variables (gender, age, economic status, marital status, level of education, and disorders for which they came to the clinic). This information was used only for the purpose of a descriptive analysis. The questionnaire and main items can be found in Table A1 (Appendix A).

### 3.3. Data Analysis

The data were analyzed by using IBM SPSS Statistics (version 23, Armonk, NY, USA). We performed the descriptive statistics (percentages, mean, and standard deviation, and correlation coefficient). To answer question 1, we applied the multiple linear regression, where the dependent variable was general satisfaction with the clinical recuperation services (SG), and the predictors were physician’s behavior (PB), the impact of interventions on the state of health (IHI), and modern medical equipment (ME). To answer question 2, we applied the logistic regression. The intention to return to the clinic was the dependent variable, and the general satisfaction with the clinical recuperation services (SG) was the predictor.

## 4. Results

### 4.1. What Was the Impact of the Physician’s Behavior, the State of Health Following the Intervention, and the Modern Medical Equipment on the General Degree of Satisfaction with the Services Offered by the Clinic?

#### 4.1.1. Descriptive Statistics

The results of the descriptive statistics analysis are presented in detail in Table 3. The surveyed patients have a high degree of satisfaction with the services offered by the clinic (SG) targeted in this study (M = 8.46, SD = 1.4) and have a high degree of satisfaction with the equipment of that clinic (ME) (M = 4.1, SD = 0.77), and essentially consider that the therapists followed the protocol and had a good interaction with the clients (PB) (M = 18.64, SD = 3.58). Although the vast majority of therapists followed the protocols and patients had a positive perception of physician’s behavior, not all patients perceived significant changes in their health status after the interventions (M = 2.31, SD = 1.25).

There are also some vulnerabilities in the behavior of therapists, such as the fact that few of them performed an initial psychic evaluation (32.4%), few discussed fears related to medical recovery (51.4%), and some therapists did not recommend exercise at home (23.1%) (Table 4).

#### 4.1.2. Regression Analysis

The simple regression analysis indicates the fact that 45.5% of the variation of the variable satisfaction with the services offered by the clinic (SG) can be explained by the physician’s behavior (PB), the impact of interventions on the state of health (IHI), and the modern equipment owned by the clinic (ME), with the model being statistically significant (F = 27.14, *p* < 0.001). The analysis of regression coefficients indicates the fact that all predictors included in the model are statistically significant (*p* < 0.05), and the most important predictor is physician’s behavior (PB) (Beta = 0.406), followed by modern medical equipment (ME) (Beta = 0.339), and in the last position, the impact of interventions on the state of health (IHI) (Beta = 0.168) (Table 5).

### 4.2. To What Extent Can the Satisfaction with the Services Offered by the Clinic Predict the Intention to Return to the Recuperation Clinic?

#### 4.2.1. Descriptive Statistics

Although patients have a reasonably high degree of satisfaction with the services of the clinic (Table 6), only 64.8% say they will definitely return to this clinic, 30.5% are not sure whether they would return or not, and 4.8% would definitely not return. In this context, we wished to see if the satisfaction with the clinical services can predict the intention to return to the clinic. For this, we performed a binomial logistic regression model.

#### 4.2.2. Regression Analysis

We performed a binomial logistic regression, where the dependent variable was the intention to return to the clinic (IRC) recoded with two categories (1 = I would return, 0 = I would not return), and the independent one, the general satisfaction with the clinical recuperation services (SG). The regression analysis indicates that the SG variable is a significant predictor (B = 1.1991, *p* < 0.01). In 99% of the cases that expressed the intention to return to the clinic, the prediction based on SG was correct, and in 80% of the cases of those who expressed the intention of not returning, the prediction was correct (Table 7).

## 5. Discussion

The data in this study reflect that satisfaction with the services offered by a medical recuperation clinic is a predictor for the intention to use its services in the future. Other studies indicate a tight relationship between these two variables [22,52]. Our study does not reflect a strong relationship. According to our model, the intention to return to a clinic is not explained only by the SG (the values of Pseudo R Square indicate different values of the extent of the variation of the variable IRC). Still, indeed, we can state that this variable has a particular impact on IRC. Probably the intention of not returning is not given necessarily by the dissatisfaction with the services, but perhaps it can be generated by the fact that the health issues were solved and there are no more reasons to return. 

Furthermore, it is interesting that the general satisfaction with the services offered by a clinic is given mainly by the way in which therapists interact with clients and less by the result in itself of the interventions. These data are in line with other studies in which the physician’s behavior factor is presented as determined or moderator in the evaluation of health services [52,53,54,55,56,57]. Consequently, physician’s behavior (PB) is the most important variable for making predictions regarding the satisfaction with the services offered by the clinic both in ordinary time and in COVID-19 time. Modern equipment is a factor that contributes to the satisfaction with a clinic, but if the behavior of the therapist is as expected by clients, this can generate a high degree of satisfaction. Manzoor suggests that the physician’s behavior moderates significantly the effect of healthcare services on patient satisfaction and it can influence the gap between the various medical services and patient satisfaction [57]. Some studies indicate that there is an association between the physician–patient relationship and the results reported by patients. For this reason, physicians have to be aware of the way in which their behavior influences the satisfaction of the patient; administrative staff should take into account any prior relationship between patient and physician when they assess the results reported by the patient [54].

It is important that physicians observe some procedures and explain them to patients. Thus, patients have the feeling that they participate in the treatment process and have full knowledge of the facts. For instance, patients have suggested the fact that few times an initial psychological evaluation was performed, therapists sometimes neglected to tell them about a series of exercises to do at home or to discuss with patients the fears related to medical recuperation. Other studies have suggested that the first session has a major role in establishing a good relationship between patient and therapist [20,58]. In this case, there is room for improvement, and a better awareness of this by physiotherapists from the respective clinic would surely lead to a higher degree of satisfaction.

It is interesting that the impact of therapeutic interventions on patients does not represent the most important factor, as would have been expected. The results are similar to those of another study showing that overall patient satisfaction is significantly but weakly correlated with the treatment effects [15]. Where the differences were higher between the state of health before the intervention and that after the intervention, the individuals offered higher scores for satisfaction with the services of the clinic. However, there also were patients who did not have significant health issues who appealed to the clinical services, and the improvement did not have high values (the difference between the initial evaluation and the final one) because the health issues were not significant, and then the IHI variable did not capture a high considerable difference between the two scores. In other words, where there existed significant differences in their state of health after the intervention of therapists, the scores were higher. Still, there also were persons who gave higher scores even if they did not have significant changes. Another study shows that, after rehabilitation treatments, patients could be dissatisfied with treatment but satisfied with the care received [59]. These persons have the capacity of being aware that the respective clinic offered them all that was possible, but there is a series of disorders where significant changes cannot occur in a relatively short-term.

Another essential element is the clinical context, and in this case, we considered modern equipment as the main indicator. As it results from our study, modern equipment contributes to an increase in the degree of satisfaction, but it is not necessarily a determining factor for the intention to return to the clinic. The intention to return to a clinic is given by the satisfaction with the clinical services, and this is provided especially by the behavior of therapists. This is in line with other studies that indicate the fact that the clinical context has a major role in establishing an optimal relationship between patient and therapist [20,58,60]. Thus, modern equipment can be an auxiliary factor that can contribute to the state of patients and an increase in confidence in the medical staff.

This study has a series of limits. The size of the research sample and the method of its selection does not allow the generalization of the obtained results. Thereby, the study reflects the opinions of patients of only one medical recuperation clinic, where patients with simple disorders took part in the study. Patients in other clinics, with more serious disorders, may have different opinions about the factors that influence their level of satisfaction.

Moreover, we could not test in this research a series of variables that characterize the behavior of physicians mentioned in the literature.

The number of independent variables used is definitely too small to precisely explain the shaping of the phenomenon of patient satisfaction.

Subsequent studies could target an integrated model that could explain the satisfaction with the services of a clinic and the intention to return to a clinic. Moreover, new methods of analysis could be used to establish satisfaction degrees and categories of clients. The use of index variables that measure the loyalty of customers in the marketing area could be constructed on such patients, and based on them, typologies of customers could be created. Predictions can be made regarding subsequent behavior [61]. The role of such variables is necessary for establishing a strategy of developing loyalty and attracting new customers.

## 6. Conclusions

There are several implications for companies that provide medical recuperation services in Romania. The main contribution of this paper is to show that, in the COVID-19 context, the influence of physician behavior on patient satisfaction is stronger than the influence of others variables, such as the results of medical interventions or modern equipment. Considering the results of other studies conducted under ordinary condition, we can say that the physiotherapist’s behavior has an essential role in determining the patients’ satisfaction both in ordinary time and in COVID-19 time. Thus, the importance of the patient–therapist relationship is confirmed in the Romanian context. Another noteworthy result is the relationship between satisfaction and physician behavior and patients’ intention to return to the clinic. Patients have the intention to return depending on the satisfaction with the services, and the satisfaction with the services is provided by the patient–therapist relationship that is built from the first session. Enhanced attention should be paid to this and to observing protocols. More than that, an adaptability model of physicians, depending on the needs of patients, deserves to be targeted. Patients have mentioned that the therapist did not discuss many fears related to medical recuperation; therefore, a more open attitude of Romanian therapists is necessary and useful.

The study was conducted in unusual conditions immediately after leaving the quarantine when the clinic reopened to the public. The opinions of these patients are significant. These patients represent the category that needed special treatment and allowed itself to pay for the treatment. These results are essential for studying the behavior of patients who resorted to the clinic’s services in pandemic conditions. The study is unique and highlights that patient satisfaction is directly related to the behavior of the physiotherapist. The study results help medical staff in the field to know the opinions of patients who pay extra for these medical services in pandemic conditions. The study can be used as a method of evaluation in other medical services. For doctoral students and researchers in the field, the study makes significant contributions and can generate research directions in other medical specialties. The study also provides essential information for those who want to create and deliver medical services in private clinics.

The study did not determine all potential characteristics which may have had effects on patient satisfaction. The analysis should be performed in several private clinics.

## Figures and Tables

**Table 1:** Sociodemographic characteristic of respondents.

Characteristic	Category	Count	Percentage
Gender	Female	61	58.1%
	Male	44	48.9%
Education	Elementary school	9	8.6%
	High school	50	47.6%
	Higher education	46	43.8%
Age	19–40 years	23	21.9%
	41–60 years	33	31.4%
	over 60 years	49	46.7%
Marital status	Married/consensual union	73	69.5%
	Single	15	14.3%
	Widowed	15	14.3%
	Divorced	2	1.9%
Income	Low (less than 1500 ron)	19	18.1%
	Medium (between 1500 and 3000 ron)	73	69.5%
	High (greater than 3000 ron)	13	12.4%

**Table 2:** The distribution of patients depending on the disorders for which they presented to medical recuperation services.

Disorder	Count	Percentage
Tetraparesis	4	3.7%
Paraparesis	3	2.8%
Lumbar discopathy	27	25.7%
Ankle sprain	4	3.7%
CDL Spondylosis	17	16.2%
Ankylosing spondylosis	1	1%
Disc protrusion	2	1.9%
Scoliosis	2	1.9%
Collateral ligament injury	1	1%
Torn meniscus	1	1%
Fractured kneecap	1	1%
Gonarthrosis	10	9.5%
Subpatellar bursitis	1	1%
Hemiparesis	8	7.6%
Herniated disc	7	6.6%
Epicondylitis	1	1%
Hip congenital sprain	1	1%
Facial palsy	1	1%
Bimalleolar fracture+fibula	1	1%
Broken arm	1	1%
Hip algofunctional sequelae	1	1%
Shoulder sprain	1	1%
PSH	4	3.7%
Osteoarthritis	4	3.7%
Sciatic pain	1	1%
Total	105	100%

**Table 3:** Descriptive statistics and correlation coefficient.

Variables	Minimum	Maximum	Mean	SD	1	2	3
1. General satisfaction with the clinical recuperation services (SG)	1	10	8.46	1.40			
2. Physician’s behavior (PB) summative index	0	22	18.64	3.58	0.571 *		
3. Impact of interventions on the state of health (IHI) (HBI–HAI)	0	6	2.31	1.25	0.332 *	0.239 *	
4. Modern medical equipment (ME)	1	5	4.1	0.77	0.614 *	0.370 *	0.174

Note: *n* = 105. * Correlations are significant at the 0.01 level (2-tailed).

**Table 4:** Descriptive statistics—physician’s behavior (PB).

Items	Category	Percent
1. The therapist performed an initial motor evaluation for you.	The therapist did this as he should	85.7%
2. The therapist carried out an initial psychic evaluation for you.	The therapist did this as he should	32.4%
3. The therapist answered the questions you asked him.	The therapist did this as he should	81.9%
4. The therapist discussed with you the fears related to medical recovery.	The therapist did this as he should	51.4%
5. The therapist offered advice through which deficiencies can be remedied.	The therapist did this as he should	82.9%
6. The therapist empathized with you and understood your suffering.	The therapist did this as he should	81%
7. The therapist offered you the attention and respect due to a patient.	The therapist did this as he should	89.5%
8. The therapist offered you advice to remediate deficiencies.	The therapist did this as he should	82.9%
9. The therapist corrected your moves if they were wrong.	The therapist did this as he should	82.9%
10. The therapist presented a series of exercises at home.	The therapist did this as he should	76.9%
11. The therapist showed interest and got involved in solving/improving the state of my health.	The therapist did this as he should	84.8%

**Table 5:** Multiple linear regression.

Variables	B	SE	Beta	T	Sig.
Constant	3.089	0.634		4.871	0.000
PB	0.138	0.028	0.406 **	4.930	0.000
IHI	0.165	0.075	0.168 *	2.191	0.031
ME	0.604	0.143	0.339 **	4.212	0.000

Dependent variable: General satisfaction with clinical recuperation services (SG) R2 = 0.45, F = 27.14, *p* < 0.001 * *p* < 0.05, ** *p* < 0.001.

**Table 6:** Descriptive statistics—IRC variables.

Variables	Category	Percent
The intention to return to the clinic (IRC).	Yes, definitely	64.8%
	Maybe	30.5%
	I would not return	4.8%

**Table 7:** Binomial logistic regression.

Variables	B	SE	Exp(B)	Wald	Sig.
Constant	−10.636	4.088		6.770	0.003
SG	1.991	0.669	7.320 *	8.846	0.009

Dependent variable: The intention to return to the clinic (IRC). Nagelkerke ^2^ = 0.721, Cox&Snel R ^2^ = 0.229, * *p* < 0.01.

## Data Availability

Data will be available at Transilvania University institutional repository, http://aspeckt.unitbv.ro/jspui/ (accessed on 1 May 2021).

## Appendix A

**Table A1:** Measurement.

Concepts	Items
General satisfaction with the clinical recuperation services (SG)	On the while, how satisfied are you with the services received in this clinic? Give a mark from 1 to 10, where 1 not satisfied at all, 10 very satisfied
Physician’s Behavior (PB)	(2—The therapist did this as he should 1—The therapist did this to a certain extent 0—The therapist did not do this)
	The therapist performed an initial motor evaluation for you.
	2. The therapist carried out a psychic initial evaluation for you.
	3. The therapist answered the questions you asked him.
	4. The therapist discussed with you the fears related to medical recovery.
	5. The therapist offered advice through which deficiencies can be remedied.
	6. The therapist empathized with you and understood your suffering.
	7. The therapist offered you the attention and respect due to the patient.
	8. The therapist offered you advice to remediate deficiencies.
	9. The therapist corrected your moves if they were wrong.
	10. The therapist presented a series of exercises at home.
	11. The therapist showed interest and got involved in solving/improving the state of my health.
Changes in the state of health after an intervention (CHI)	How would you evaluate your state of health before coming to the medical recuperation center? (HBI)Give a mark from 1 to 10, where 1 very poor, 10 very good
	How would you evaluate your state of health after coming to the medical recuperation center? (HAI) Give a mark from 1 to 10, where 1 very poor, 10 very good
modern medical equipment (ME)	How satisfied are you with modern equipment? (5-point Likert scale, where 1 = very dissatisfied, 5 = very satisfied)
the intention to return to the clinic (IRC)	Would you return to the medical recuperation clinic? (2—certainly yes, 1—probably, 0—certainly no)
